# An Unusual Case of *Citrobacter koseri* Infection During Pregnancy at 27 Weeks: A Case Report and Literature Review

**DOI:** 10.1002/ccr3.72342

**Published:** 2026-03-25

**Authors:** Mario Assenza, Michele Caramia, Jacopo Wabersich

**Affiliations:** ^1^ Università degli studi di Padova, Dipartimento di Salute della Donna e del Bambino Padova Italy; ^2^ Unità operativa di ginecologia e Ostetricia, ospedale di Mirano‐Dolo, azienda ULSS 3 Serenissima Venice Italy

**Keywords:** antibiotic therapy, antimicrobial stewardship, *Citrobacter koseri*, fetal outcomes, meningoencephalitis, neonatal sepsis, pregnancy, vertical transmission

## Abstract

Early prenatal screening at 26 + 6 weeks detected *Citrobacter koseri* vaginal colonization in an asymptomatic high‐risk gravida. Culture‐guided IV ceftazidime (susceptible per CLSI testing) eradicated the pathogen without maternal/fetal compromise. Multidisciplinary management prevented vertical transmission, neonatal CNS complications, and yielded a healthy term delivery (Apgar 9/10). Emphasizes susceptibility‐directed antimicrobial stewardship for optimal outcomes in rare pregnancy infections.

## 1 Introduction

*Citrobacter koseri* is a Gram‐negative, facultative anaerobic bacillus belonging to the family Enterobacteriaceae [1]. While recognized as an opportunistic pathogen, *C. koseri* infections during pregnancy are uncommon but clinically significant because of their potential to cause severe neonatal complications, including sepsis and meningoencephalitis [2, 3]. These infections pose a notable risk for fetal morbidity and mortality, particularly when vertical transmission occurs from mother to fetus [4, 5]. Recent epidemiological data describe clusters of *C. koseri* infections associated with neonatal intensive care units and outbreaks in maternity wards [1]. These observations underscore the need for improved antenatal screening protocols and rapid management strategies tailored to prevent vertical transmission and its consequences. From a microbiological standpoint, *C. koseri* possesses virulence factors that facilitate invasion and persistence within the central nervous system, contributing to the high incidence of neonatal brain abscesses and meningitis observed in infected neonates [6, 7]. These neuropathogenic mechanisms, combined with the challenges of early diagnosis in pregnant women who may be asymptomatic, significantly complicate clinical management. The antimicrobial resistance profile of *C. koseri* strains has evolved, with increasing reports of resistance to commonly used antibiotics such as amikacin and ampicillin, thereby limiting therapeutic options [8]. Susceptibility testing remains essential for guiding effective antibiotic therapy, particularly in maternal–fetal cases where drug safety profiles must be balanced with infection control imperatives.

## 2 Case History

**FIGURE 1:**
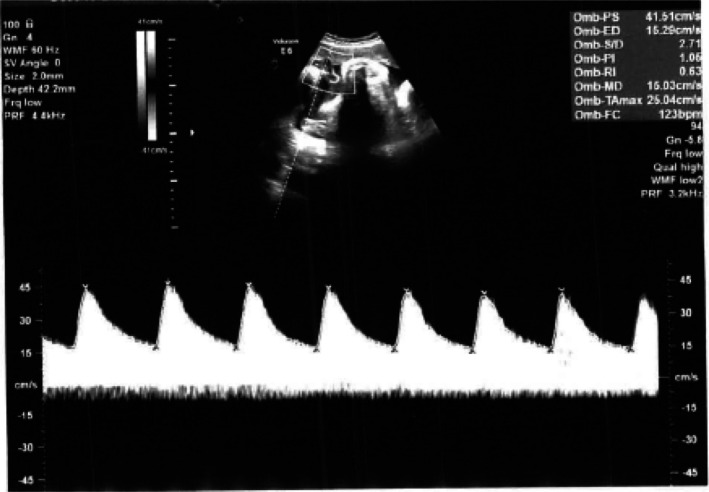
Ultrasound performed with a transabdominal probe shows a fetal umbilical artery pulsatility index of 1.06, demonstrating that good fetal tone and relative well‐being in the womb are maintained.

A 30‐year‐old woman, gravida 3 para 0, presented with a history of two prior first‐trimester spontaneous miscarriages, for which she had received prophylactic vaginal progesterone in the current pregnancy until the end of the first trimester. Her current pregnancy was otherwise uneventful, with standard prenatal screening including a negative noninvasive prenatal test (NIPT) for chromosomal abnormalities. Morphological ultrasonography revealed an amniotic band, although the patient remained asymptomatic. In view of the patient's obstetric history and the finding of cervical shortening, an additional vaginal swab was scheduled at 26 weeks of gestation. At 21 + 2 weeks of gestation, a vaginal swab had detected Ureaplasma spp. and Candida spp., which were promptly treated with appropriate antimicrobial and antifungal therapy [9]. Follow‐up swabbing at 26 + 6 weeks unexpectedly revealed asymptomatic vaginal colonization with *Citrobacter koseri* , a rare pathogen during pregnancy associated with a risk of vertical transmission and neonatal sepsis if left untreated [4, 5]. There were no elevated inflammatory markers, systemic symptoms, or sonographic evidence of ascending infection. The patient was immediately admitted for inpatient management, and an infectious diseases specialist was consulted. Given the history of miscarriage around 20 weeks and a cervical length of 25 mm on ultrasound, the multidisciplinary team decided to err on the side of caution, opting for hospitalization, close observation, and intravenous rather than oral antibiotic therapy. This conservative approach was chosen despite the absence of specific guideline recommendations for hospitalization and IV antibiotics in the setting of asymptomatic vaginal colonization with *C. koseri* . The decision was jointly made by the attending obstetrician, the head of the gynecology and obstetrics unit, the infectious diseases consultant, and the physician responsible for the high‐risk pregnancy clinic. Intravenous ceftazidime (1 g three times daily) was initiated based on antimicrobial susceptibility testing. During hospitalization, the patient remained hemodynamically stable and asymptomatic with no signs of chorioamnionitis or premature rupture of membranes. Strict isolation protocols were implemented to minimize the risk of nosocomial infection. Fetal well‐being was monitored by ultrasound, confirming normal hemodynamics and good fetal tone (Figure 1).

After 7 days of treatment, the patient was discharged with a comprehensive follow‐up plan, including serial fetal monitoring and repeat vaginal cultures to eradicate the pathogen. Given the history of early miscarriages and homozygosity for the MTHFR mutation, she was discharged on low‐dose aspirin (one tablet daily). At 37 + 3 weeks of gestation, following premature rupture of membranes, she underwent an uncomplicated vaginal delivery with a first‐degree vaginal–perineal tear requiring suturing. The male infant weighed 2488 g and had Apgar scores of 9 at 1 min, with an umbilical arterial pH of 7.18 and base excess of −6.4. He was immediately placed in skin‐to‐skin contact with his mother. The neonate underwent a complete workup for early‐onset sepsis, including blood and urine cultures, all of which were negative. Neuroimaging, including transfontanellar ultrasound and brain MRI, excluded central nervous system lesions. Neurological follow‐up at 6 and 12 months documented normal development without deficits.

## 3 Differential Diagnosis, Investigation, and Treatment

The identification of *Citrobacter koseri* was confirmed by culture using standard microbiological techniques. Antimicrobial susceptibility testing was performed according to Clinical and Laboratory Standards Institute (CLSI) guidelines to determine the most appropriate therapeutic agents. The *C. koseri* isolate was susceptible to third‐generation cephalosporins (ceftazidime, cefepime, cefotaxime) and fluoroquinolones (ciprofloxacin, levofloxacin). Additional susceptibility was observed to carbapenems (imipenem, meropenem, ertapenem), colistin, tigecycline, gentamicin, piperacillin‐tazobactam, and trimethoprim‐sulfamethoxazole. Resistance was documented to amikacin, ampicillin, ceftolozane‐tazobactam, and tobramycin. This susceptibility profile is consistent with previous reports describing variable resistance patterns among *C. koseri* strains [10]. The choice of intravenous ceftazidime was guided by susceptibility results and its established efficacy against Enterobacteriaceae, combined with an acceptable safety profile during pregnancy. The decision to use intravenous antibiotic therapy, as discussed above, was considered appropriate for this high‐risk scenario. Decision‐making in favor of IV therapy was particularly prudent given the prior miscarriages around 20 weeks and the cervical length of 25 mm on ultrasound, despite the absence of specific guidelines for asymptomatic *C. koseri* colonization. Multidisciplinary input (head of the obstetrics and gynecology unit, infectious diseases specialist, and high‐risk pregnancy clinic) supported a cautious approach to reduce the risk of ascending infection and preterm premature rupture of membranes (PPROM).

Post‐discharge, daily low‐dose aspirin was continued because of MTHFR homozygosity. Repeat vaginal cultures confirmed eradication of *C. koseri* , in keeping with antimicrobial stewardship principles in the context of an organism capable of acquiring plasmid‐mediated resistance (Table 1).

**TABLE 1:** Antimicrobial susceptibility testing results—Antibiogramma.

Antibiotic	Result (R/S/I)	MIC (μg/mL)
Amikacin	R	16
Ampicillin	R	> 8
Cefepime	S	< = 1
Cefotaxime	S	< = 1
Ceftazidime	S	< = 1
Ceftazidime/Avibactam	S	< = 2
Ceftolozane/Tazobactam	R	4
Ciprofloxacin	S	0.25
Colistin	S	< = 2
Ertapenem	S	< = 0.06
Gentamicin	S	< = 2
Imipenem	S	< = 1
Levofloxacin	S	< = 0.5
Meropenem	S	< = 0.12
Piperacillin/Tazobactam	S	< = 4
Tigecycline	S	≤ 0.5
Tobramycin	R	> 4
Trimethoprim/Sulfamethoxazole	S	< = 2/38

*Note:* The Citrobacter koseri isolate from the vaginal swab showed sensitivity to third‐generation cephalosporins, fluoroquinolones, and carbapenems and resistance to aminoglycosides and ampicillin, confirming a typical susceptibility pattern for this species.

Abbreviations: I, Intermediate; N, no interpretation; R, Resistant; S, Sensible.

The literature emphasizes early identification and targeted antibiotic therapy based on susceptibility testing. Third‐generation cephalosporins and carbapenems are often effective, although resistance patterns vary [8, 10]. Given the organism's ability to acquire resistance genes via plasmids, ongoing microbiological monitoring is crucial for guiding both empiric and targeted therapy, especially in obstetric patients where fetal safety is paramount. In this case, intravenous ceftazidime was consistent with susceptibility results and is recognized for its efficacy against Enterobacteriaceae, including Citrobacter spp., with a favorable safety profile in pregnancy [9]. Continued surveillance after therapy confirmed eradication of the pathogen, contributing to the uneventful continuation of the pregnancy.

## 4 Conclusions and Results

This case illustrates that *Citrobacter koseri* infection in pregnancy, although rare, requires prompt recognition and multidisciplinary management. Early diagnosis combined with timely, targeted antimicrobial therapy can lead to favorable maternal and fetal outcomes, in contrast to historically poor prognoses associated with delayed or inadequate treatment. Clinicians should maintain a high index of suspicion for unusual pathogens such as *C. koseri* in pregnant women with risk factors including previous pregnancy loss or ascending genitourinary infections. The importance of culture‐driven antimicrobial stewardship is paramount in this context, particularly in light of evolving resistance patterns.

In this high‐risk case (prior miscarriages around 20 weeks, cervical length of 25 mm, MTHFR homozygosity), prudent management with IV ceftazidime—guided by multidisciplinary consultation despite the absence of specific guidelines—prevented vertical transmission. This was confirmed by eradication on follow‐up cultures and negative neonatal evaluations (blood and urine cultures, neuroimaging, and normal neurodevelopment at 12 months).

Future research should focus on establishing standardized antenatal screening protocols, refining antibiotic selection strategies, and developing preventive measures to reduce the incidence of, and improve outcomes in *C. koseri* infections during pregnancy. Historically, fetal and neonatal outcomes have often been poor in reported cases, with high rates of intrauterine death, pregnancy loss, and severe neurological sequelae. However, cases with timely detection and appropriate treatment, such as the present report, demonstrate the potential for favorable outcomes when a multidisciplinary approach and targeted antimicrobial therapy are employed. This case adds to the limited literature on successful management of *C. koseri* in pregnancy and shows that, with appropriate intervention, both maternal and fetal health can be preserved.

## 5 Discussion

The reference citations included in the case presentation are intended to contextualize our findings by relating clinical and microbiological features of this case to previously published reports. Specifically, the identification of *Citrobacter koseri* colonization in our patient mirrors descriptions in existing literature, where vertical transmission and neonatal sepsis have been documented as potential complications. This comparative perspective is further elaborated in the discussion section, where outcomes and management strategies from our case are integrated and contrasted with those of prior case reports.

*Citrobacter koseri* is a rare but clinically significant pathogen in pregnancy, with the potential to cause severe maternal–fetal complications. Its neurotropic nature is well‐documented, and neonatal infections are associated with high risks of morbidity and mortality [6, 7, 11] (Table 2).

**TABLE 2:** Summary of major case reports of *Citrobacter koseri* infection in pregnancy and neonatal period.

Author	Year	Gestational age	Fetal outcome	CNS involvement	Antibiotic resistance	Notes
Bonasoni et al. (2022) [1]	2022	2nd trimester	Fetal loss	Yes	Yes	PPROM, intrauterine infection
Agrawal et al. (2005) [3]	2005	Neonatal	Brain abscess	Yes	ND	Vertically acquired
Papasian et al. (1996) [4]	1996	Term	Neonatal sepsis	Yes	Yes	Vertical transmission confirmed
Mastrobattista et al. (1997) [5]	1997	Term	Neonatal sepsis	Yes	ND	Intra‐amniotic infection
Rodrigues et al. (2014) [6]	2014	3rd trimester	Variable outcomes	Yes	Yes	Four cases analyzed
Chan et al., (2002) [7]	2002	2nd trimester	Fetal death	Yes	ND	Two cases of fetal loss
Panek et al. (2025) [11]	2025	Neonatal	Brain death	Yes	Yes	Irreversible brain damage

Abbreviations: CNS, Central nervous system; ND, Not documented; PPROM, Preterm premature rupture of membrane.

Vertical transmission during pregnancy represents a major clinical challenge because of the risk of severe fetal and neonatal complications, including meningoencephalitis, sepsis, and adverse neurological outcomes.

The available literature, although limited, consistently shows substantial morbidity and mortality following maternal–fetal transmission, particularly in the presence of risk factors such as preterm premature rupture of membranes (PPROM) and intra‐amniotic infection. Several case reports highlight the spectrum of possible outcomes. Papasian et al. (1996) provided molecular evidence of vertical transmission resulting in neonatal sepsis, confirming maternal–neonatal linkage by ribotyping and pulsed‐field gel electrophoresis [4]. Mastrobattista et al. (1997) described intra‐amniotic infection leading to neonatal disease [5], whereas Chan et al. (2002) reported second‐trimester fetal deaths associated with *C. koseri* chorioamnionitis [7]. Agrawal et al. (2005) described a vertically acquired neonatal brain abscess [3], and Rodrigues et al. (2014) reported four cases of neonatal *C. koseri* meningitis, likely related to maternal–fetal transmission [6]. More recent reports, such as Bonasoni et al. (2022), documented second‐trimester fetal loss linked to *C. koseri* infection and PPROM [1], while Panek et al. (2025) described fatal neonatal sepsis with irreversible brain injury despite aggressive intervention [11]. Across these studies, recurring themes include central nervous system involvement, severe or fatal outcomes in the absence of rapid, targeted therapy, and the complicating role of antimicrobial resistance. Poor outcomes are often associated with delayed diagnosis, the presence of PPROM, or failure to eradicate infection before delivery. In contrast, the present case demonstrates that favorable maternal and fetal outcomes are achievable when *Citrobacter koseri* is identified early and managed with a multidisciplinary strategy. The patient received prompt inpatient care and targeted intravenous antimicrobial therapy based on susceptibility testing, resulting in the eradication of the pathogen and the prevention of neonatal infection. The absence of CNS involvement and neurological sequelae in the newborn underscores the importance of vigilance in prenatal screening, rapid pathogen identification, and culture‐guided therapy.

In this high‐risk pregnancy (prior miscarriages around 20 weeks, cervical shortening to 25 mm, and MTHFR homozygosity), the multidisciplinary decision to admit the patient and administer IV ceftazidime despite the lack of explicit guidelines for asymptomatic *C. koseri* colonization was pivotal. This cautious approach, informed by obstetric, infectious diseases, and high‐risk pregnancy specialists, mitigated the heightened risk of vertical transmission associated with patient‐specific factors. Comprehensive neonatal evaluation (negative blood, urine, and CSF cultures, normal transfontanellar ultrasonography and MRI, and normal neurodevelopment at 12 months) supports the effectiveness of this strategy and contrasts sharply with reports of devastating CNS outcomes. Together, these findings reinforce the value of culture‐guided antimicrobial stewardship, even in colonization scenarios. Overall, the literature highlights the rare but serious potential of *C. koseri* to cause vertical transmission with devastating consequences. Early diagnosis and coordinated multidisciplinary management can dramatically alter the prognosis, as illustrated by this case. Standardized screening protocols and evidence‐based therapeutic strategies are needed to better define risk factors, guide empiric therapy, and prevent adverse outcomes associated with this challenging pathogen [6, 7, 8, 11].

## Author Contributions

**Mario Assenza:** conceptualization, data curation, formal analysis, funding acquisition, investigation, methodology, project administration, resources, software, supervision, validation, visualization, writing – original draft, writing – review and editing. **Michele Caramia:** conceptualization, formal analysis, investigation, methodology, resources, validation, visualization, writing – original draft. **Jacopo Wabersich:** conceptualization, data curation, funding acquisition, project administration, supervision, validation, visualization.

## Funding

The authors have nothing to report.

## Disclosure

Human subjects: Consent was obtained or waived from all participants in this study. Written informed consent was obtained from the patient for the publication of clinical data and images. This case report was conducted in accordance with institutional guidelines and ethical standards. No formal ethics committee approval was required for this retrospective case report.

## Consent

The authors have nothing to report.

## Conflicts of Interest

The authors declare no conflicts of interest.

## Data Availability

The data that support the findings of this study are available from the corresponding author upon reasonable request.
